# Cathodic deposition of CoNi-ZIFs on molecularly imprinted polymers for synergistically enhanced electrochemical detection of rutin

**DOI:** 10.1039/d6ra09585b

**Published:** 2026-11-10

**Authors:** Rongqian Meng, Shengjuan Shao, Lijun Guo, Hong Yang

**Affiliations:** a Department of Chemistry and Chemical Engineering, Taiyuan Institute of Technology Taiyuan 030008 China

## Abstract

Rutin often coexists with other flavonoids possessing similar electroactive sites in natural plants, resulting in poor selectivity in electrochemical detection. An efficient electrochemical sensor was developed by cathodically depositing CoNi-Zeolitic Imidazolate Frameworks (ZIFs) onto molecularly imprinted polymer (MIP) films to improve the detection selectivity for rutin. The deposition conditions of the CoNi-ZIFs/MIP composite electrode were systematically optimized and the morphology, structure and electrocatalytic performance of CoNi-ZIFs/MIP composite electrodes using different deposition times were investigated. Results showed that the composite electrode presented two distinct morphologies—granular and block-like—which featured different Co/Ni atomic ratios. The electrode prepared with the deposition time of 250 s exhibited higher current, smaller charge transfer resistance, larger effective specific surface area, higher area ratios of Co^2+^/Co^3+^ and Ni^2+^/Ni^3+^, and higher content of oxidized nitrogen species, thereby enhancing electrocatalytic activity for rutin. CoNi-ZIFs incorporated onto MIP/GCE further amplified potential differences (Δ*E*) and current ratios between rutin and three structurally similar flavonoids, synergistically enhancing rutin selectivity, presumably due to their electrocatalytic discrimination of flavonoids with varied C-ring substitutions. This sensor exhibited low detection limit (*i.e.* 2.4 nM) and wide detection range (*i.e.* 0.01–1 µM and 1–100 µM). Furthermore, the sensor demonstrated good repeatability, reproducibility, and anti-interference ability, and was successfully applied for accurate determination of rutin in real Flos Sophorae Immaturus samples.

## Abstract 2

Cathodic deposition of CoNi-ZIFs onto the molecularly imprinted polymer amplified the potential differences and current ratios between rutin and three similar flavonoids, synergistically enhancing rutin detection selectivity.

## Graphical abstract

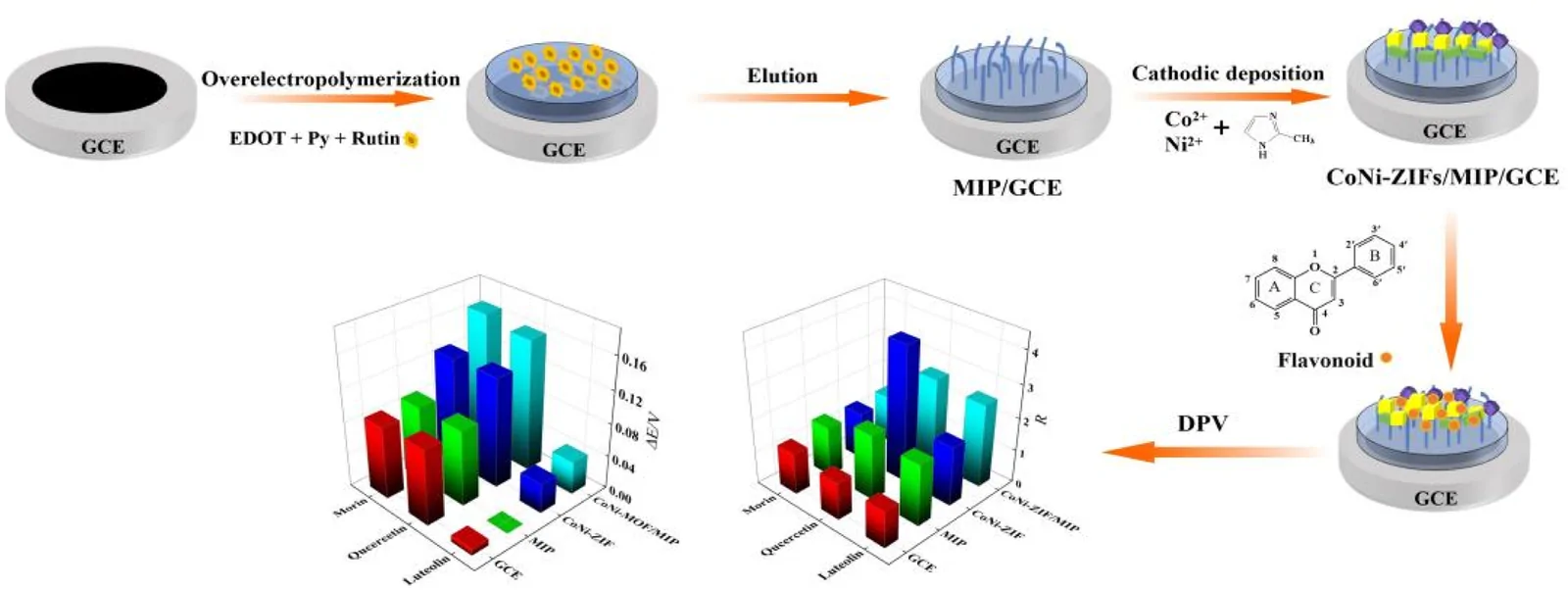

## 1. Introduction

Rutin is a naturally occurring flavonoid commonly found in a variety of vegetables, fruits, and herbs that form part of the daily human diet, and it exhibits well-defined pharmacological actions, including antioxidant, microcirculatory, antiplatelet, and neuroprotective effects.^1^ Various methods have been developed for rutin detection, including high-performance liquid chromatography (HPLC),^2^ capillary electrophoresis (CE),^3^ ultraviolet-visible spectrophotometry,^4^ and electrochemical analysis.^5^ Among these, electrochemical methods are particularly notable for their operational simplicity, rapid analysis, high sensitivity, and low detection limits.^6,7^ Rutin can be electrochemically detected *via* the oxidation of its phenolic hydroxyl groups on the B-ring (primary site) and A-ring (secondary site).^8,9^ This process forms quinone-like intermediates, resulting in electron transfer and the generation of characteristic current peaks, whose potential and intensity can be monitored for analysis.^10–12^

Rutin frequently coexists with other flavonoids like quercetin, luteolin, and morin in plant sources such as Flos Sophorae Immaturus (FSI), buckwheat and Pueraria roots.^2,13–15^ When these coexisting flavonoids (such as luteolin and quercetin) possess the same electroactive sites as rutin, they exhibit similar electrochemical responses on the electrode surface, including overlapping oxidation peak potentials and similar current magnitudes.^16^ This poses a key challenge for its selective electrochemical determination.

Molecularly imprinted polymer (MIP) technology, combined with electrochemical sensors, serves as an effective recognition strategy to significantly enhance the selectivity of electrochemical sensors.^17,18^ In our previous study, the introduction of molecular imprinting technology into the electrochemical detection system significantly enhanced the electrode's recognition capability for rutin, yielding at least a threefold increase in the differential pulse voltammetry (DPV) current compared to other structurally similar flavonoids. However, the peak potential shift for rutin and its analogues remained minimal—a notable limitation for selective analysis.^19^

In flavonoids, whose structural core is 2-phenylchromone (Fig. 1), the coordination of metal ions with specific groups such as the 3-OH and 4-C=O on the C-ring, the 3,4-catechol moiety on the B-ring, or the 5-OH (A-ring) and 4-C=O (C-ring) induces structural destabilization, thereby facilitating oxidation and resulting in a decrease in the peak potential.^20,21^ The differences in metal coordination sites within flavonoid compounds will have different effects on the changes in peak potential.^21^ For example, under acidic conditions, trivalent metal ions such as Al^3+^ and Fe^3+^ can coordinate with the 3-OH and 4-C=O groups on the C-ring as well as the 3,4-catechol unit on the B-ring in flavonoids to form stable complexes, which subsequently induce negative shifts in peak potential accompanied by the decrease in peak current.^22^ In contrast, besides the 3-OH and 4-C=O groups on the C-ring, divalent metal ions like Co^2+^and Ni^2+^ preferentially bind to the 5-OH group on the A-ring and the 4-C=O group on the C-ring, while showing negligible coordination with the 3,4-catechol moiety on the B-ring, thereby offering greater advantages in electrochemical detection.^23^ Thus, it is promising to enhance the selectivity of electrochemical rutin detection by incorporating these metal ions like Co^2+^and Ni^2+^ into the molecular imprinting polymer (MIP) electrode material. However, when directly immobilized on the electrode surface, these metal ions tend to be unstable, prone to leaching and aggregation.

**Fig. 1:**
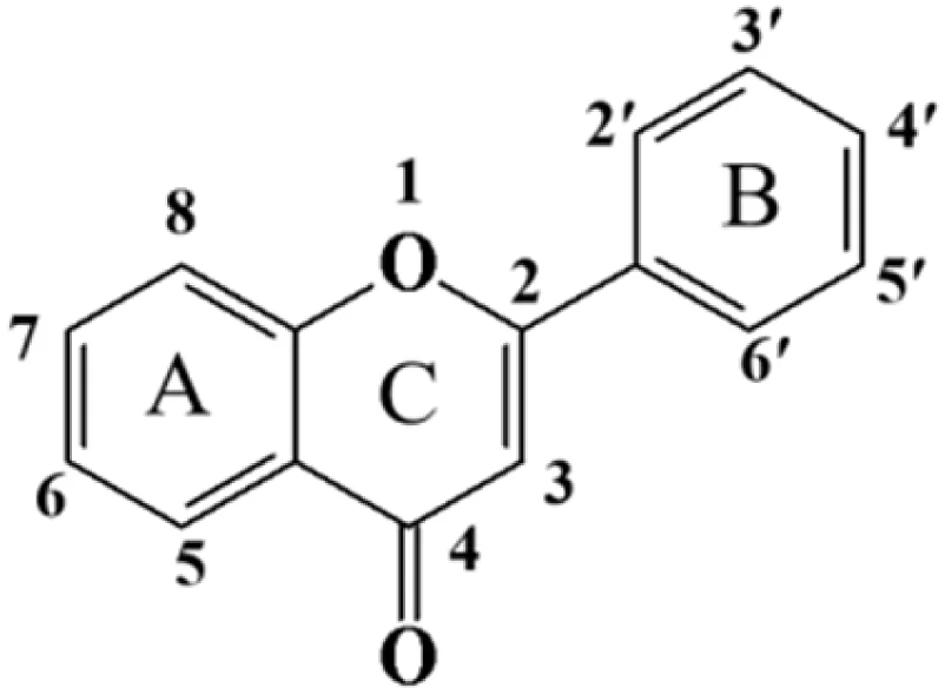
The structural nucleus of flavonoids.

Zeolitic imidazolate frameworks (ZIFs), a type of metal–organic frameworks (MOFs) containing metal ions and imidazole-based organic ligands, have shown great promise for extraction,^24^ adsorption^25^ and sensing.^26,27^ However, the electrochemical applications of ZIFs are severely limited by their inherent drawbacks, including low electrical conductivity and physical stability. Previous studies have demonstrated that bimetallic ZIFs give rise to new structural defects, leading to increased exposure of active sites and unsaturated metal coordination, resulting in improved electrocatalytic performance and enhanced binding capacity for target molecules.^28^ Common methods for preparing ZIFs-based electrode materials include hydrothermal synthesis,^29^ dip/spin coating,^30^ secondary growth,^31^ and electrodeposition.^32^ Electrodeposition method has attracted increasing attention due to its rapid processing, mild reaction conditions, and tunable thickness and morphology. Among these, cathodic deposition enables the direct synthesis of MOFs on the electrode surface without sacrificing the working electrode, offering simpler operation and broader applicability.^33^

Herein, the MIP was electropolymerized on a glassy carbon electrode (GCE) using pyrrole and 3,4-ethylenedioxythiophene as bifunctional monomers and rutin as the template, following our previously reported procedure.^19^ Then, CoNi-ZIFs layer was grown on MIP surface by cathodic deposition methods to obtain CoNi-ZIFs/MIP/GCE, and the deposition parameters were systematically optimized. The effects of deposition time on the electrocatalytic performance of CoNi-ZIFs/MIP toward rutin were investigated from the perspective of morphology, structure and electrochemical characterization. Subsequently, by analyzing the differential pulse voltammetry (DPV) potential differences (Δ*E*) and current ratios of four flavonoid compounds, the selective recognition capability of the composite electrode for rutin was systematically evaluated, and the underlying structure–activity relationship was further explored. The analytical performance of the sensor was assessed *via* experiments on repeatability, reproducibility, and anti-interference capability. Furthermore, the sensor was employed for the determination of rutin in FSI. This study introduces a novel, simple and feasible approach for constructing highly selective electrochemical sensors for rutin detection.

## 2. Experimental

### 2.1. Materials and instruments

The experimental materials and instruments were listed in the SI Section 2.1.

### 2.2. Fabrication of CoNi-ZIFs/MIP/GCE

The electrode was polished according to the method previously reported in the literature. The MIP and non-molecularly imprinted polymer (NIP) were prepared following the method reported in our previous work^19^ and were described in the Section 2.2 of SI. The CoNi-ZIFs were deposited on MIP/GCE by cathodic deposition method. Firstly, 2 mmol of Co(NO_3_)_2_·6H_2_O, 2 mmol of Ni(NO_3_)_2_·6H_2_O, and 16 mmol of 2-methylimidazole (2-mIM) were dissolved in 20 mL of 95% ethanol solution (with 5% H_2_O). Then, after ultrasound for 5 min, the electrodeposition solution was obtained. MIP/GCE was immersed in this electrodepotion solution. A constant cathodic potential of −2.0 V *vs.* saturated calomel electrode (SCE) was applied to grow CoNi-ZIFs on MIP/GCE with electrodeposition time of 250 s. The preparation process of the composite electrode was shown in Scheme 1. As a control, CoNi-ZIFs/NIP/GCE was prepared by the same method, but with NIP in place of MIP. In order to investigate the growth kinetics process of CoNi-ZIFs, the modified electrodes prepared with deposition times of 200 s and 300 s were designated as CoNi-ZIFs-200 s/MIP/GCE and CoNi-ZIFs-300 s/MIP/GCE, respectively.

**Scheme 1:**
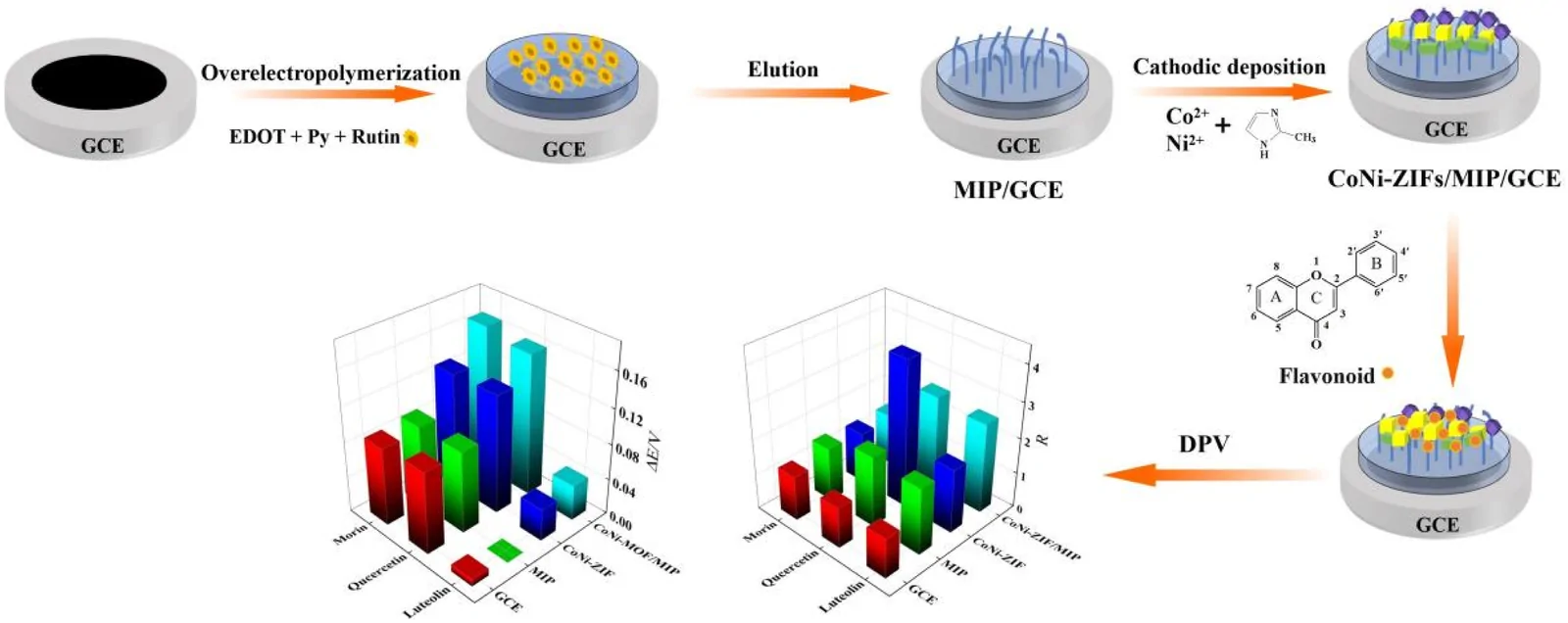
Illustration of the construction process of CoNi-ZIFs/MIP/GCE. (△*E* = *E*_rutin_ − *E*_other flavonoids_, *R* = *I*_rutin_/*I*_other flavonoids_).

### 2.3. Electrochemical measurements

The electrocatalytic performance and electrocatalytic selectivity of the electrode material toward rutin were investigated by Cyclic Voltammetry (CV) and DPV. Electrochemical impedance spectroscopy (EIS) was measured in a solution containing 5 mM [Fe(CN)_6_]^3−/4−^ and 0.1 M KCl solution over the frequency range of 0.01 Hz to 100 kHz with an AC amplitude of 5 mV. Chronocoulometry (CC) was performed in 0.10 M KCl containing 1.0 mM K_3_[Fe(CN)_6_]. DPV measurements were recorded in the potential window of 0.1–0.6 V with a pulse amplitude of 50 mV, a pulse width of 50 ms, a step potential of 5 mV, and a pulse period of 500 ms. Prior to the DPV tests, the electrodes were incubated in the rutin solution for 5 min to allow for target rebinding.

### 2.4. Preparation of real samples

The dried FSI were ground and sieved through 40-mesh screen. The resulting powder, stored in a desiccator at ambient temperature, served as the test sample. Approximately 0.10 g of the sample was ultrasonically extracted with 90 mL of methanol at 40 °C for 50 min in a sealed vessel. After filtration, 4 mL aliquot of the filtrate was transferred into 25 mL volumetric flask and diluted to the mark with pH 4.2 acetic acid buffer. The resulting solution was then used for the subsequent determination of rutin. The recovery evaluation was conducted using the standard addition method, based on three independent extractions and measurements at each concentration level (*n* = 3). The DPV peak current corresponding to rutin in the extract was recorded using CoNi-ZIFs/MIP/GCE. The recovery was calculated to investigate the analytical method accuracy. The high-performance liquid chromatography (HPLC) method was employed to validate the accuracy of the established method, and the specific experimental conditions were detailed in SI.

## 3. Results and discussion

### 3.1. Optimization of the preparation conditions of CoNi-ZIFs/MIP/GCE

The preparation conditions of the CoNi-ZIFs/MIP/GCE were optimized by examining the impacts of three key parameters, including the Co^2+^/Ni^2+^ molar concentration ratio, deposition potential, and deposition time on the rutin DPV peak current (Fig. S1). With the total molar concentration of metal ions fixed at 0.2 mol L^−1^, bimetallic ZIFs exhibited higher peak current toward rutin than their monometallic counterparts, confirming the synergistic electrocatalytic effect arising from the Co–Ni bimetallic combination. The peak current reached its maximum at a Co^2+^ : Ni^2+^ molar ratio of 1 : 1. As the cathodic potential increased, the peak current of rutin at the composite electrode gradually increased. This behavior was likely attributable to the enhanced enrichment of metal ions on the MIP surface and the electrogeneration of more OH^−^ ions at higher potentials, which facilitated ligand deprotonation and thereby promoted the cathodic synthesis of ZIFs. The peak current at −2.0 V reached maximum. However, further increase in potential led to a decline in current, presumably due to excessive ZIF deposition that increased electrode thickness and thus elevated electron transfer resistance. The deposition time influenced the peak current response of the composite electrode toward rutin, with the maximum peak current achieved at 250 s. To further elucidate the growth process of CoNi-ZIFs, the effects of deposition time on the electrochemical performance, morphology, and structure of the CoNi-ZIFs/MIP electrode were systematically investigated.

### 3.2. Electrochemical analysis

As illustrated in Fig. 2A, DPV measurements were conducted on various electrodes in a 0.1 mM rutin acetate buffer solution (ABS, pH 4.2). Direct deposition of CoNi-ZIFs onto the bare GCE surface resulted in smallest DPV peak current for rutin, which may be attributed to the absence of sufficient metal nucleation sites on GCE, leading to easy aggregation and detachment of the CoNi-ZIFs,^34^ as well as the poor electrical conductivity of organic ligands—collectively resulting in limited electrocatalytic activity toward rutin. When single-metal ZIFs (Co-ZIFs and Ni-ZIFs) were individually deposited on the MIP/GCE surface, the DPV peak currents improved moderately. The influence of different modification sequences of CoNi-ZIFs and MIP was also investigated. The CoNi-ZIFs/MIP/GCE exhibited a higher peak current for rutin than the MIP/CoNi-ZIFs/GCE, achieving the highest current response among all tested electrodes. This indicated that the combination of bimetallic CoNi-ZIFs with the conductive MIP substrate synergistically boosted the electrocatalytic performance for rutin. Furthermore, regardless of whether the single-metal or bimetallic ZIFs were formed on MIP, their peak potentials for rutin shifted to varying extents toward lower values, suggesting that the introduction of ZIFs made the oxidation reaction of rutin easier to occur.

**Fig. 2:**
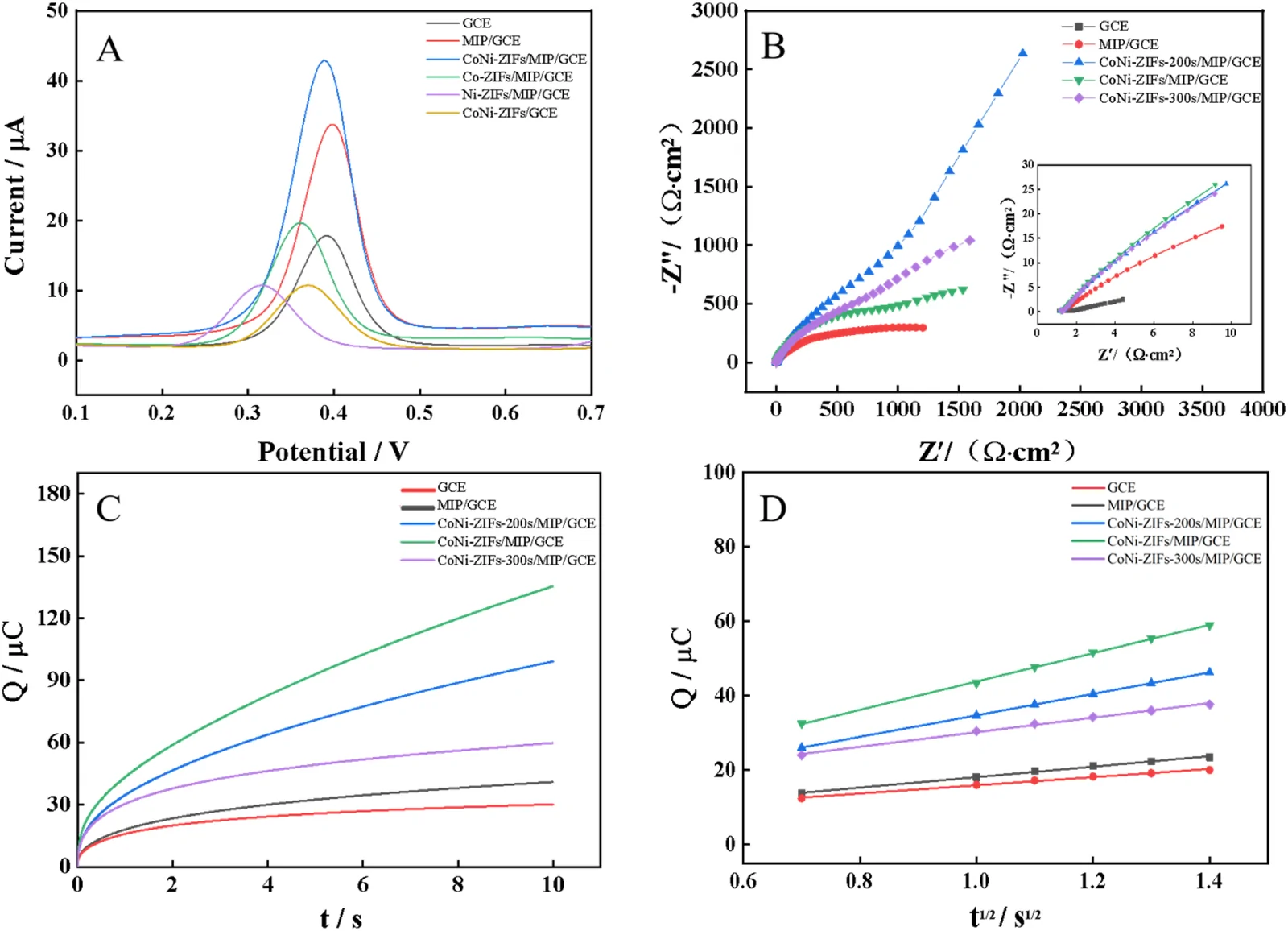
(A) The DPV curves of different electrode materials in a 0.1 mM rutin ABS (pH 4.2); (B) EIS of different electrode materials in 5 mM [Fe(CN)_6_]^3−/4−^ and 0.1 M KCl solution; (C) *Q*–*t* and (D) *Q*–*t*^1/2^ curves of different electrode materials in 0.10 M KCl solution containing 1.0 mM K_3_[Fe(CN)_6_].

The EIS measurements for different electrodes were shown in Fig. 2B, and the fitted equivalent circuits along with the corresponding resistance data derived from ZSimpWin software were provided in Fig. S2 and Table S4. As shown, the solution resistance (*R*_s_) and high-frequency film resistance (*R*_1_) remained relatively stable, suggesting that the overall interfacial resistance was primarily dominated by the charge-transfer resistance (*R*_ct_). The GCE exhibited the smallest *R*_ct_, followed by the MIP. After CoNi-ZIFs deposition, *R*_ct_ increased, likely due to the poor intrinsic conductivity of ZIFs, which hindered probe charge transfer. Notably, the *R*_ct_ values followed a non-linear trend: CoNi-ZIFs/MIP (5698 Ω cm^2^) > CoNi-ZIFs-300 s/MIP (4760 Ω cm^2^) > CoNi-ZIFs-200 s/MIP (4113 Ω cm^2^). This likely reflected the morphological evolution of the ZIFs layers: the thin film at 200 s may have allowed easier probe access, the thick and loose film at 250 s presumably caused the longest diffusion path, and the dense crystalline film at 300 s could have facilitated electron transfer through the dense crystalline film.

The CC analysis method was employed to investigate the chrono-coulometric curves of different composite electrode materials in Fig. 2C. Based on the Anson equation,^35^ the effective specific surface area (*A*) was calculated using the formula as follows:
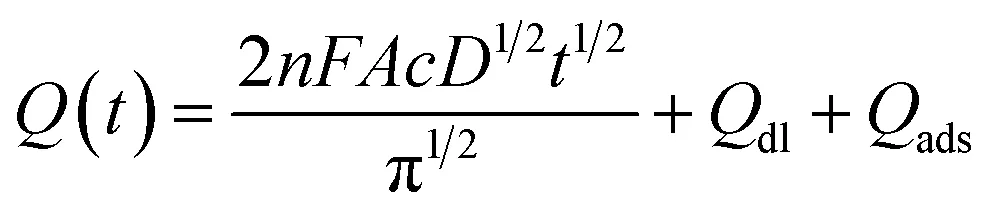
In the equations, *Q*_ads_ denotes the Faraday charge, *D* is the diffusion coefficient, *n* refers to the number of electrons transferred. For 1 mM K_3_[Fe(CN)_6_] solution, the diffusion coefficient *D* is 7.60 × 10^−6^ cm^2^ s^−1^, and *n* equals 1. From Fig. 2D, the slopes of *Q*–*t*^1/2^ for GCE, MIP/GCE, CoNi-ZIFs-200 s/MIP/GCE, CoNi-ZIFs/MIP/GCE and CoNi-ZIFs-300 s/MIP/GCE were 1.10 × 10^−5^ C (*R*^2^ = 0.995), 1.40 × 10^−5^ C (*R*^2^ = 0.997), 2.88 × 10^−5^ C (*R*^2^ = 0.999), 3.81 × 10^−5^ C (*R*^2^ = 0.999) and 1.95 × 10^−5^ C (*R*^2^ = 0.999), respectively. According to formula, the corresponding effective specific surface areas (*A*) were calculated as 0.036, 0.046, 0.094, 0.126 and 0.064 cm^2^. The *A* value of the MIP/GCE increased slightly compared with that of the bare GCE. Notably, further deposition of CoNi-ZIFs onto the MIP/GCE surface led to the marked increase in *A* value. Specifically, The CoNi-ZIFs/MIP/GCE prepared at 250 s showed approximately 1.34- and 1.97-fold higher *A* values than those at 200 s and 300 s, respectively, indicating larger specific effective area.

To gain insight into the electrochemical reaction mechanism of rutin, CV experiments were performed at scan rates ranging from 0.05 to 0.35 V s^−1^ in 0.1 mM rutin solution (ABS, pH 4.2). As illustrated in Fig. 3A and B, the peak currents increased with rising scan rates (*ν*), suggesting strong linear relationship. The corresponding linear regression equations were *I*_pa_ = 38.21*ν* + 0.72 (*R*^2^ = 0.993) and *I*_pc_ = −35.79*ν* + 1.36 (*R*^2^ = 0.997), indicating the typical adsorption-controlled process.

**Fig. 3:**
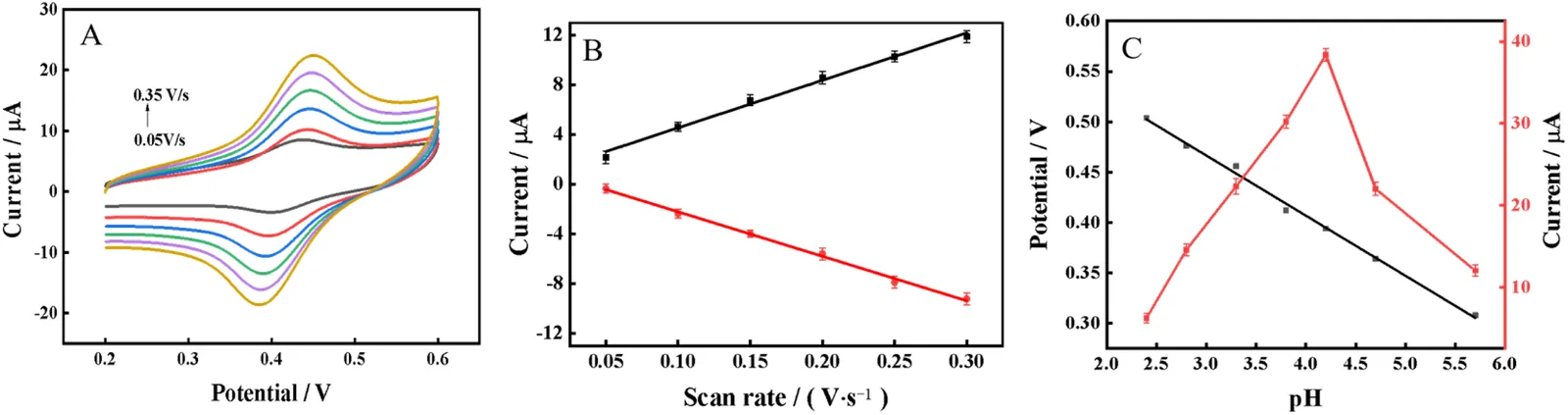
(A) The CVs curves of 0.1 mM rutin on CoNi-ZIF/MIP/GCE in pH 4.2 ABS at different scan rates; (B) linear relationship between the *I*_pa_ and *I*_pc_ with the scan rate; (C) effect of pH value on the *E*_pa_ and *I*_pa_.

The electrocatalytic performance of 0.1 mM rutin on the CoNi-ZIFs/MIP/GCE was examined over pH range of 2.0 to 6.0 by DPV methods. As depicted in Fig. 3C, the negative shift in the peak potential was observed upon raising the pH, which was interpreted as evidence of proton involvement in the electrochemical reaction. The oxidation peak potential was linearly related to pH according to the equation *E*(V) = −0.0569pH + 0.6456 (*R*^2^ = 0.9967), indicating that the redox reaction of rutin at the CoNi-ZIFs/MIP/GCE involved equal numbers of electrons and protons. The highest current signal was recorded at pH 4.2 in ABS; therefore, this pH value was chosen for all subsequent experiments.

### 3.3. Structure characterization

The crystalline structures of the as-prepared CoNi-ZIFs at different deposition times were investigated by XRD. As illustrated in Fig. 4A, the diffraction peaks at 2*θ* values of 10.6°, 13.0°, 18.3°, 22.3°, 24.8°, 33.1° correspond to the (002), (112), (222), (114), (134), (235) planes, which were consistent with the reported papers.^28,36,37^ This result confirmed that the CoNi-ZIFs catalyst was prepared successfully. The lower crystallinity of CoNi-ZIFs-200 s/MIP and CoNi-ZIFs/MIP compared with CoNi-ZIFs-300 s/MIP may be attributed to two possible factors: the hydrogen evolution reaction (HER) during electrodeposition, which could disrupt the ordered ZIF framework,^38^ and incomplete coordination between metal ions and ligands at shorter deposition times. Both effects were alleviated with prolonged deposition time, thereby leading to enhanced crystallinity.

**Fig. 4:**
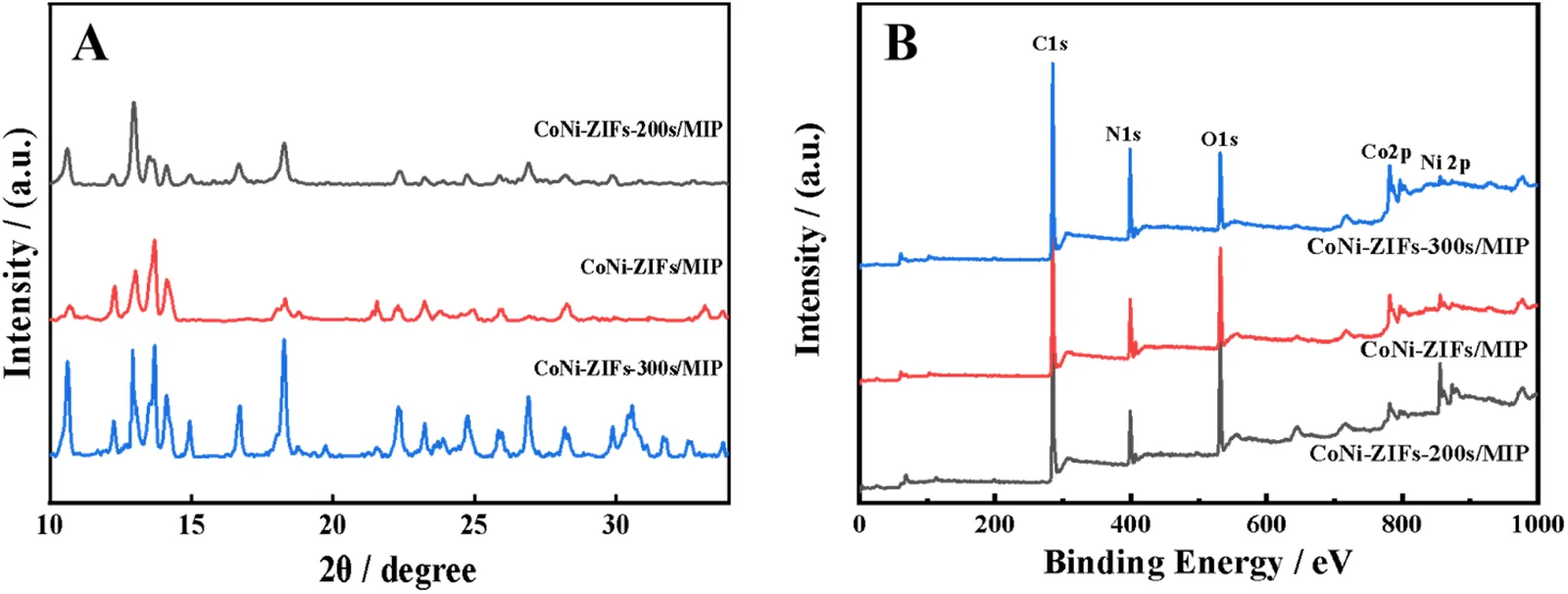
(A) XRD and (B) XPS spectra of the CoNi-ZIFs-200 s/MIP, CoNi-ZIFs/MIP and CoNi-ZIFs-300 s/MIP.

XPS full-scan spectra of CoNi-ZIFs-200 s/MIP, CoNi-ZIFs/MIP and CoNi-ZIFs-300 s/MIP were shown in Fig. 4B. As the deposition time increased, the Co : Ni atomic ratios reached 0.53, 2.87, and 5.27, indicating that Co^2+^ participated in coordination more readily than Ni^2+^. The high-resolution XPS spectra of Co 2p, Ni 2p, and N 1s in the CoNi-ZIFs/MIP composites were subjected to peak deconvolution and presented in Fig. 4. The Co 2p spectra of three composites were deconvoluted into eight peaks ascribed Co^3+^ 2p_1/2_, Co^3+^ 2p_3/2_, Co^2+^ 2p_1/2_ and Co^2+^ 2p_3/2_, and four satellite peaks.^39–41^ The Binding energy and corresponding areas were presented in the Table S1. With increasing deposition time, the Co^2+^/Co^3+^ area ratios of the three as-prepared composite material were 0.41, 0.95, and 0.71 (Table S1), respectively. Notably, the CoNi-ZIFs prepared at 250 s exhibited higher ratio of Co^2+^ to Co^3+^, which correspondingly promoted the electron transfer during oxygen reaction process.^42,43^

As shown in Fig. 5, the Ni 2p XPS spectrum can be deconvoluted into eight peaks corresponding to Ni^3+^ 2p_1/2_, Ni^3+^ 2p_3/2_, Ni^2+^ 2p_1/2_, Ni^2+^ 2p_3/2_ and four satellite peaks.^30,34^ The Binding energy and corresponding areas were presented in the Table S2. The Ni^2+^/Ni^3+^ area ratios for CoNi-ZIFs-200 s/MIP, CoNi-ZIFs/MIP, and CoNi-ZIFs-300 s/MIP were determined to be 2.05, 2.48, and 2.25, respectively (Table S2). The larger ionic radius of Ni^2+^ (0.069 nm) compared to Ni^3+^ (0.056 nm) endowed Ni^2+^ with better electrocatalytic ability.^44^ So the higher Ni^2+^ content in the CoNi-ZIFs/MIP was beneficial for the enhancement of their electrocatalytic capability. Four peaks were observed in the deconvoluted N 1s spectrum (Fig. 4), and were assigned to pyridinic-N (∼398.5 eV), metal-N (∼399.2 eV), pyrrolic-N (∼400.4 eV), and oxidized N/nitrate species (∼406.8 eV), respectively.^36,45^ The metal-N component confirmed imidazole N coordinating to Co/Ni centers in the ZIF framework, while pyridinic-N and pyrrolic-N were generally associated with edge and vacancy/defect configurations.^46^

**Fig. 5:**
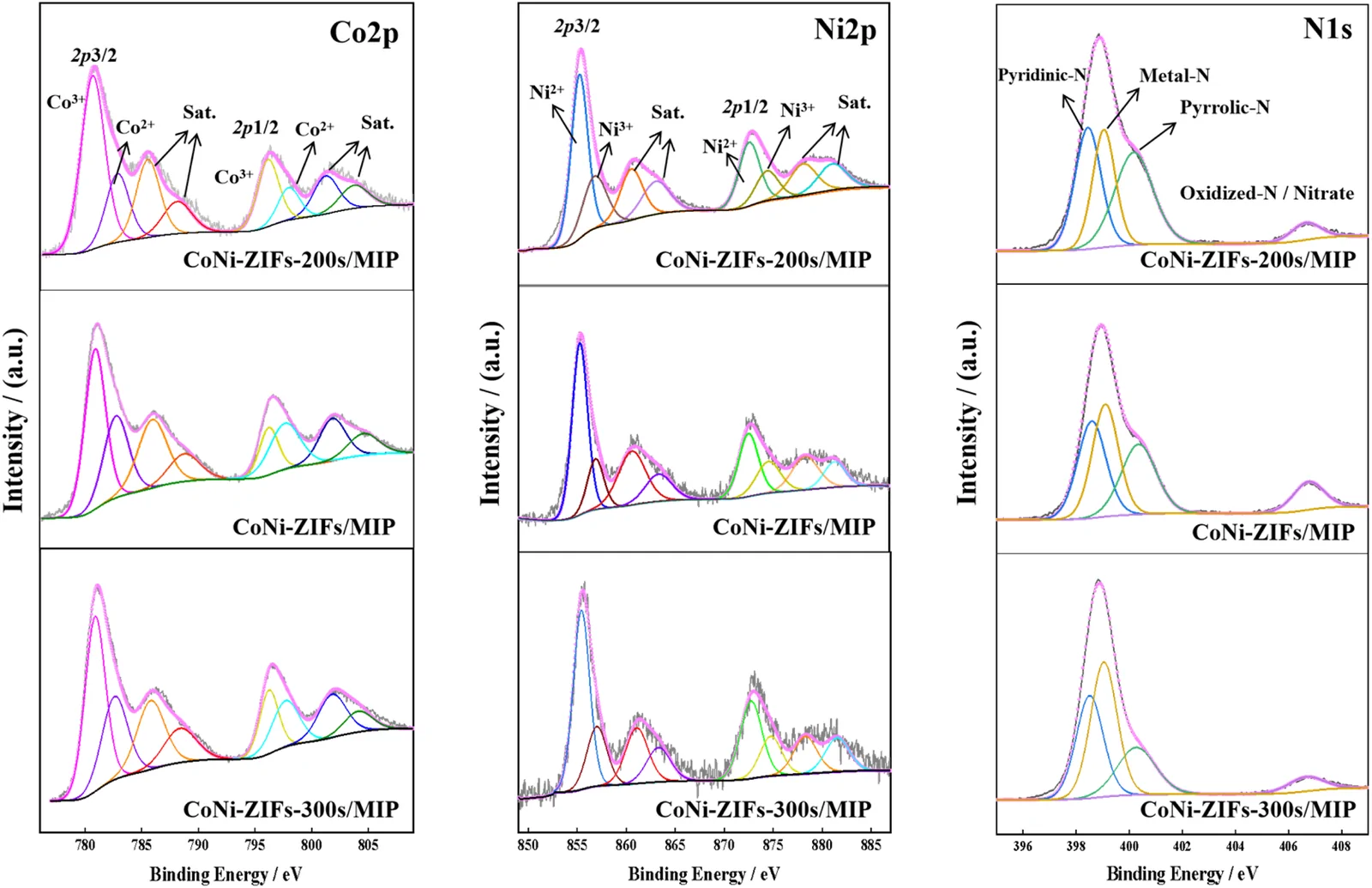
XPS spectra of Co 2p, Ni 2p, and N 1s of the CoNi-ZIFs-200 s/MIP, CoNi-ZIFs/MIP and CoNi-ZIFs-300 s/MIP.

The morphologies of MIP, CoNi-ZIFs/MIP at different deposition time were observed by SEM in Fig. 6. The MIP exhibited 3D nanorod morphology as our previous report^19^ (Fig. 6A). Upon the growth of CoNi-ZIFs on the MIP surface, two distinct morphologies—namely granular and block-like structures—were observed (Fig. 6B). With increasing deposition time, the CoNi-ZIFs layer became progressively denser with reduced porosity (Fig. 6C). The morphologis of CoNi-ZIFs/MIPs with different magnifications were observed (Fig. 6D–F), and high-magnification images of CoNi-ZIFs revealed that the polyhedral granular structures mainly consisted of cubic and dodecahedral geometries (Fig. 6F). The dodecahedral particles corresponded to the typical ZIF-67 framework, whereas the cubic particles emerged upon heterometallic Ni doping, which was consistent with previous reports.^47^Fig. 6G, H and N–R showed the elemental mapping images and atomic ratio of granular and block-like CoNi-ZIFs/MIP, respectively. It was observed that the atomic ratios of C, N, and O were similar across both morphologies. Notably, the Co : Ni atomic ratio in the granular regions was about 2 : 1, while that in the block-like regions approached about 1 : 1.5. This suggested that Co and Ni ions were incorporated into the ZIF framework at different doping levels, potentially facilitating the formation of defects and unsaturated metal coordination sites.^45,47^

**Fig. 6:**
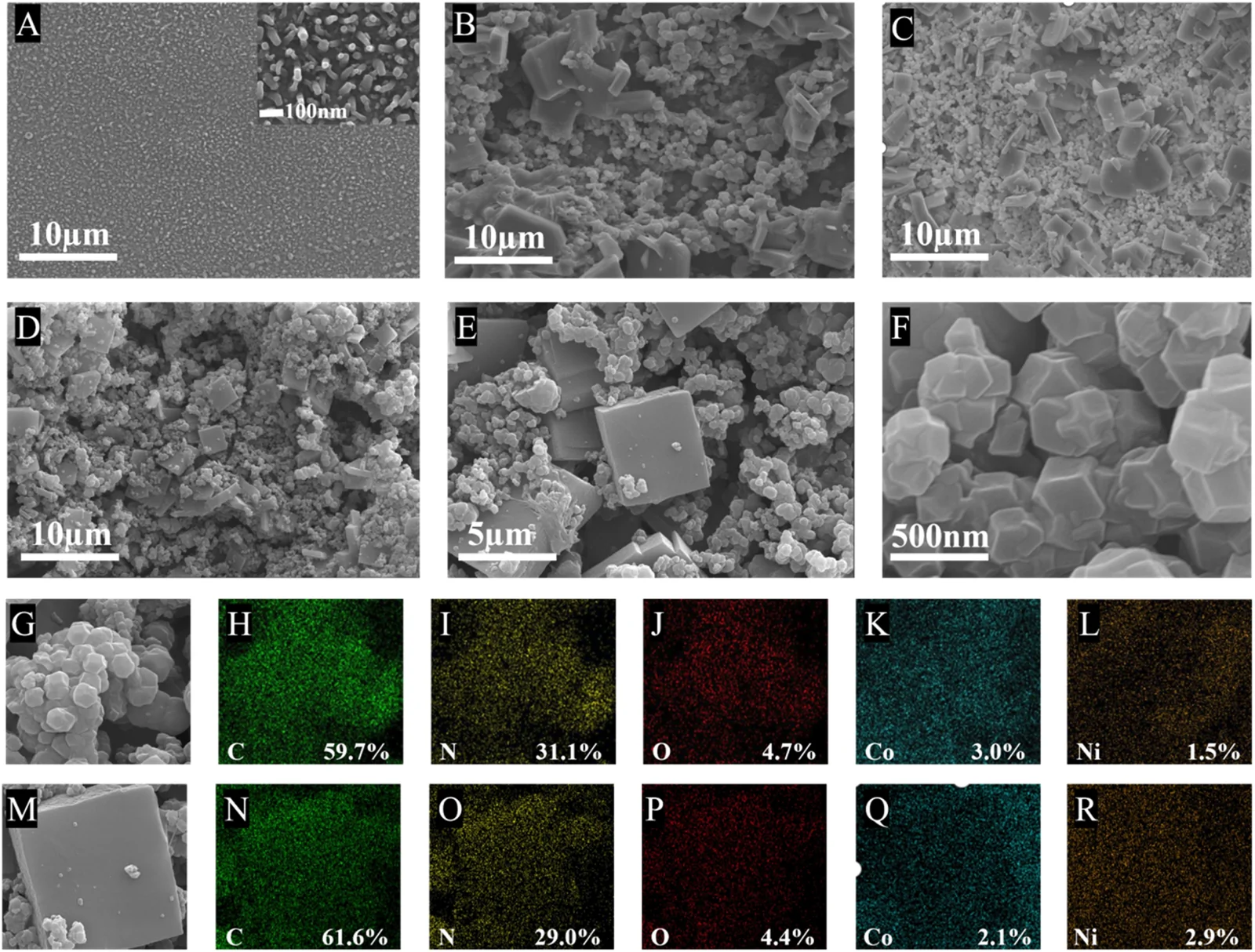
SEM images of MIP (A), CoNi-ZIFs-200 s/MIP (B), CoNi-ZIFs-300 s/MIP (C), and CoNi-ZIFs/MIP (D) with 5000 magnification, CoNi-ZIFs/MIP with 20 000 (E) and 50 000 (F) magnification; elemental mapping images of the granular (G and H–L) and blocky (M and N–R) morphologies of CoNi-ZIFs/MIP.

### 3.4. Selectivity analysis

#### 3.4.1. Potential difference (Δ*E*) analysis

As shown in the Table 1 and Fig. 7A–F, the DPV results of rutin and three other structurally similar flavonoids on six fabricated electrodes were recorded to investigate the electrode's selectivity. The structures and possible metal interaction sites of rutin, luteolin, qucercetin and morin were shown in Fig. 7G.^22,23^ The Δ*E* value, defined as the difference between the peak potential of rutin and those of the other flavonoids measured on the same electrode, was used to evaluate the electrode's selectivity toward rutin.

**Table 1:** DPV data analysis of four flavonoids (0.1 mM) in pH 4.2 ABS on four different electrodes^a^

	GCE		MIP		NIP		CoNi-ZIFs		CoNi-ZIFs/MIP		CoNi-ZIFs/NIP	
	*E*/V	*I*/µA	*E*/V	*I*/µA	*E*/V	*I*/µA	*E*/V	*I*/µA	*E*/V	*I*/µA	*E*/V	*I*/µA
Rutin	0.392	15.75	0.392	29.65	0.392	10.65	0.386	8.894	0.388	38.71	0.388	17.01
Luteolin	0.384	13.94	0.392	15.73	0.396	11.49	0.350	4.890	0.352	15.25	0.366	15.75
Qucercetin	0.300	13.67	0.300	14.81	0.296	13.95	0.252	2.161	0.278	14.55	0.284	15.17
Morin	0.304	12.54	0.300	19.48	0.300	14.74	0.254	6.215	0.282	26.94	0.280	19.61

a Δ*E* is defined as the potential difference between rutin and other flavonoids measured on the same electrode.

**Fig. 7:**
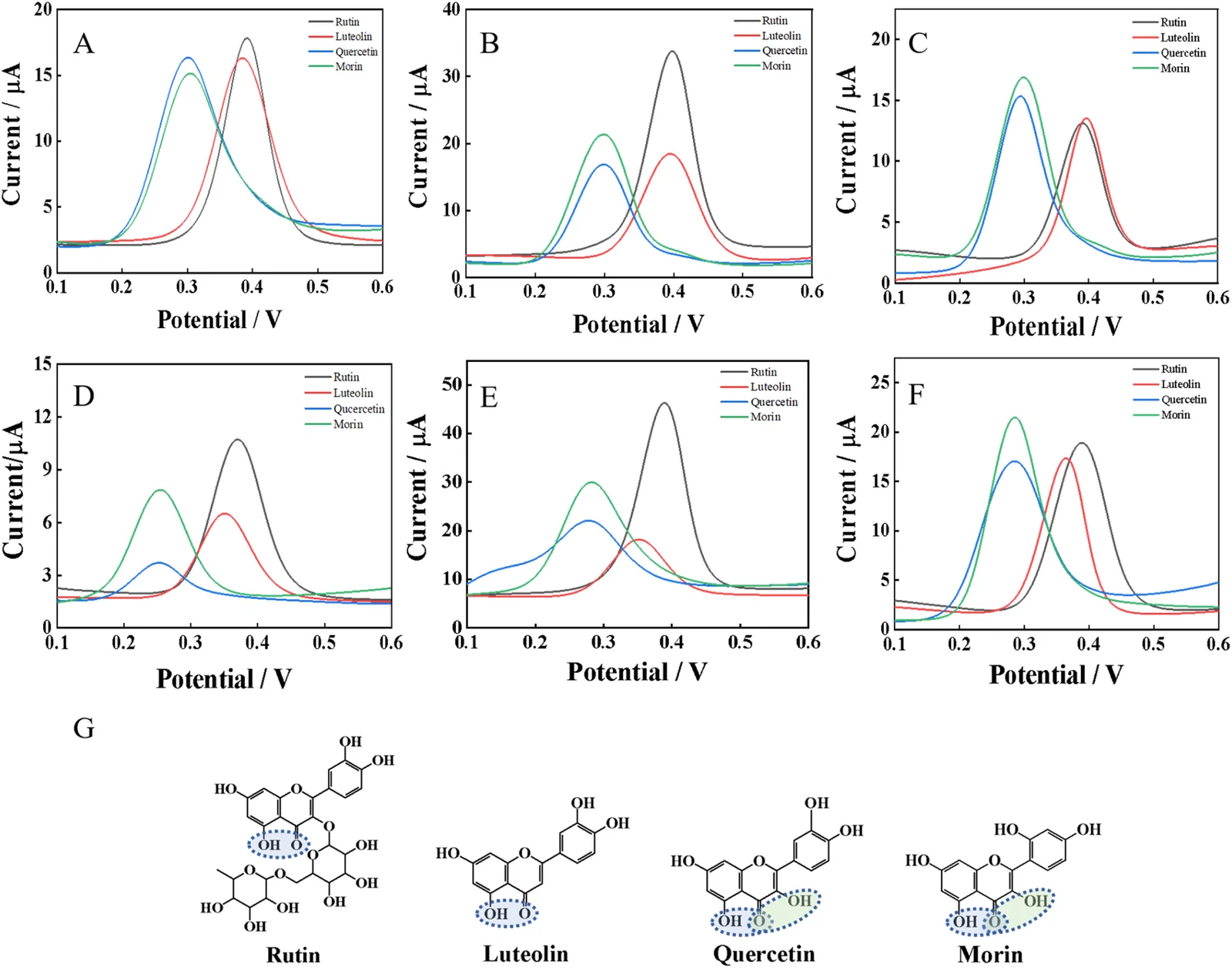
DPV curves of four flavonoids at the concentration of 0.1 mM in pH 4.2 ABS on GCE (A), MIP/GCE (B), NIP/GCE (C), CoNi-ZIFs/GCE (D), CoNi-ZIFs/MIP/GCE (E) and CoNi-ZIFs/NIP/GCE (F); the structures and the possible metal interaction sites of four flavonoids (G).

On the bare GCE, the oxidation peak potentials for both quercetin and morin on the GCE were lower than those for rutin and luteolin, showing the Δ*E* of about 0.09 V (0.092 V and 0.088 V, respectively, Fig. 7A). This difference was attributed to the presence of the 3-hydroxyl group on the C-ring, which facilitated oxidation of the phenolic hydroxyl group located on the B-ring.^16,48^ Modification of the GCE with MIP or NIP did not substantially alter the peak potentials, except for luteolin, which showed a relatively larger positive shift of 0.008 V on MIP and 0.012 V on NIP compared with that on the bare GCE (Fig. 7B and C).

In contrast, all four flavonoids exhibited negatively shifted peak potentials on the CoNi-ZIFs/GCE to varying degrees, accompanied by the increase in Δ*E* (Fig. 7D). The larger Δ*E* values, *i.e.*, 0.134 V and 0.132 V, were observed for quercetin and morin, respectively, followed by luteolin with Δ*E* value of 0.036 V, demonstrating that CoNi-ZIFs possessed stronger differentiated ability toward these structurally similar flavonoids. This phenomenon may be attributed to the interaction of the unsaturated bimetallic sites in CoNi-ZIFs with the 3-OH on the C ring/5-OH on the A ring and 4-C=O groups on the C ring of flavonoids, which modified electron density and accelerated electron transfer.^22^ For luteolin, the absence of the 3-OH group on the C-ring may diminish its charge transfer capability.^49^ For rutin, the rutinoside substituent at the 3-position introduced steric hindrance effect that impeded metal center access to the 5-OH and 4-C=O groups. Collectively, the structural variation at the 3-position of the C-ring served as the key structural feature of the observed differential electrocatalytic behavior.

When CoNi-ZIFs was deposited onto the MIP/GCE surface, the Δ*E* values for quercetin and morin (0.110 V and 0.106 V, respectively) (Fig. 7E) were smaller than those observed with CoNi-ZIFs alone. This may be because using MIP as the substrate for CoNi-ZIFs deposition weakened the interaction between the metal centers in CoNi-ZIFs and the active groups of the flavonoids. The CoNi-ZIFs/NIP/GCE exhibited comparable or slightly smaller Δ*E* values (0.022 V for luteolin, 0.104 V for quercetin) compared with the CoNi-ZIFs/MIP/GCE (Fig. 7F). This indicated that the absence of imprinting cavities in NIP weakened its ability to differentiate among these structurally similar flavonoids.

#### 3.4.2. DPV current ratio analysis

The rutin oxidation peak currents on the different electrodes were investigated to evaluate electrode selectivity. On the MIP/GCE, the rutin peak current was 1.88, 2.00, and 1.52 times those of luteolin, quercetin, and morin, respectively. In contrast, on the NIP/GCE, the rutin peak current was even lower than those of luteolin, quercetin, and morin, with ratios of 0.93, 0.76, and 0.72, indicating that NIP lacks specific recognition cavities for rutin. On the CoNi-ZIFs/GCE, the peak currents of the four flavonoids were relatively low, likely due to the limited electrocatalytic activity of ZIFs itself. Nevertheless, rutin still showed the highest peak current, possibly owing to the selective adsorption of ZIFs toward its multiple hydroxyl groups.

Notably, compared with MIP/GCE, CoNi-ZIFs/MIP/GCE exhibited higher current ratios for rutin over luteolin and quercetin (2.54 and 2.66, respectively), while the morin ratio slightly decreased from 1.52 to 1.43. Although the ratio for morin decreased, the absolute current difference between rutin and morin actually increased. These results suggested that the composite electrode still provided stronger electrocatalytic selectivity for rutin. In comparison, the CoNi-ZIFs/NIP/GCE gave ratios of only 1.08, 1.12, and 0.87 for luteolin, quercetin, and morin, respectively; morin even exceeded rutin in peak current. Therefore, the composite electrode fabricated by using MIP as the substrate to induce CoNi-ZIFs deposition exhibited better selectivity toward rutin than other tested electrodes.

These results demonstrated that the composite electrode exhibited an overall superior performance for rutin detection compared to structurally similar interferents. Although a few exceptions showed slight decreases relative to single electrode material, the current ratios and potential separation were enhanced in most cases.

Furthermore, luteolin (Lu), quercetin (Qu) and morin (Mo) were added to ABS containing 0.1 mM rutin (Ru) respectively to evaluate the selectivity of the CoNi-ZIFs/MIP/GCE (Fig. S3A–C). The DPV results showed that peaks of Lu, Qu, Mo and Ru were found at potentials of 0.346, 0.278, 0.286 and 0.388V, respectively, which were essentially consistent with those obtained in single component detection. In addition, these structurally similar compounds in mixed solution have little interference with the peak current detection of rutin, further demonstrating the desirable selectivity of the composite electrode toward rutin.

### 3.5. Reproducibility, repeatability, stability and anti-interference

Reproducibility tests conducted with five identically prepared electrodes yielded similarly consistent responses with the RSD of 1.90% (Fig. 8A), indicating excellent fabrication reproducibility. Within-electrode repeatability was evaluated by five consecutive measurement cycles, which gave an RSD of 4.51%, and the current decreased to 91.0% of its initial value. This decline likely resulted from strong rutin adsorption and aggregation at the electrode interface, which possibly blocked active sites and impeded electron transfer (Fig. 8B). Moreover, more than 91.6% of the initial sensor response was maintained after being stored at 4 °C for seven days, revealing the acceptable stability of the sensor. Furthermore, to evaluate the anti-interference capability of the CoNi-ZIFs/MIP/GCE, the peak current response toward 0.1 mM rutin was examined in the presence of various interferents, including structural analogues (luteolin, quercetin, morin) at the equivalent concentration (0.1 mM) to rutin, as well as inorganic salts (KCl, MgSO_4_, CaCl_2_, NaNO_3_) and organic small molecules (glucose, sucrose, citric acid, uric acid) at the 50-fold concentration (5 mM) relative to rutin. As illustrated in Fig. 8C, the variation in the peak current induced by these interfering substances remained below 5%, demonstrating the excellent anti-interference property of the fabricated sensor.

**Fig. 8:**
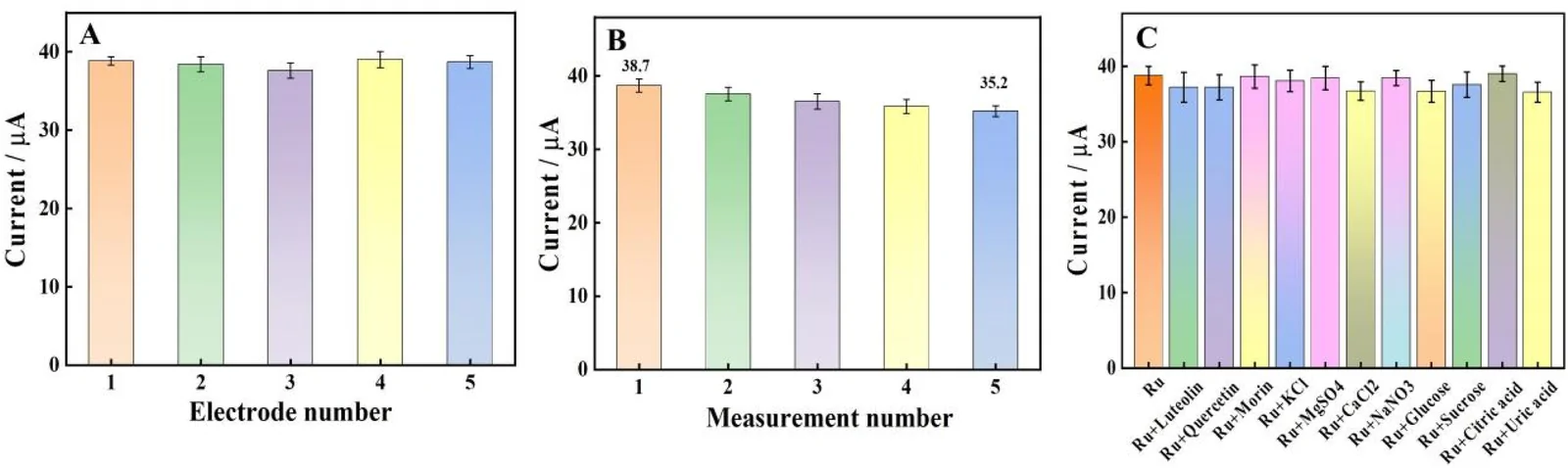
(A) DPV responses of five independently prepared electrodes; (B) DPV responses of the CoNi-ZIFs/MIP/GCE for five repetitive measurements on the same electrode; (C) oxidation peak current of 0.1 mM rutin containing 5 mM metal ions and organic small molecules.

The interference concentrations of structurally similar flavonoids were further examined. As shown in Fig. S3 and Table S5, when the concentrations of luteolin, quercetin, and morin were increased to 2.5 times that of rutin, the relative errors (RE) of the rutin current remained below 5% (ranging from 0.77% to 4.37%). When the concentrations were further increased (to 3-fold for luteolin and 5-fold for quercetin and morin), the RE values rose to approximately 10%. This indicated that the method exhibited acceptable tolerance towards structurally similar flavonoids, and the interference effect exhibited the concentration-dependent manner.

### 3.6. Quantitative analysis of rutin on CoNi-ZIFs/MIP/GCE

To assess the detection performance of the sensor, DPV signals corresponding to various concentrations of rutin on the CoNi-ZIFs/MIP/GCE were recorded under the optimal experimental conditions (Fig. 9). The linear regression equations were as follows:*I* (µA) = 1.87*C* (µM) + 0.52, *R*^2^ = 0.993 (0.01 to 1 µM),*I* (µA) = 0.37*C* (µM) +1.81, *R*^2^ = 0.992 (1 to 100 µM).

**Fig. 9:**
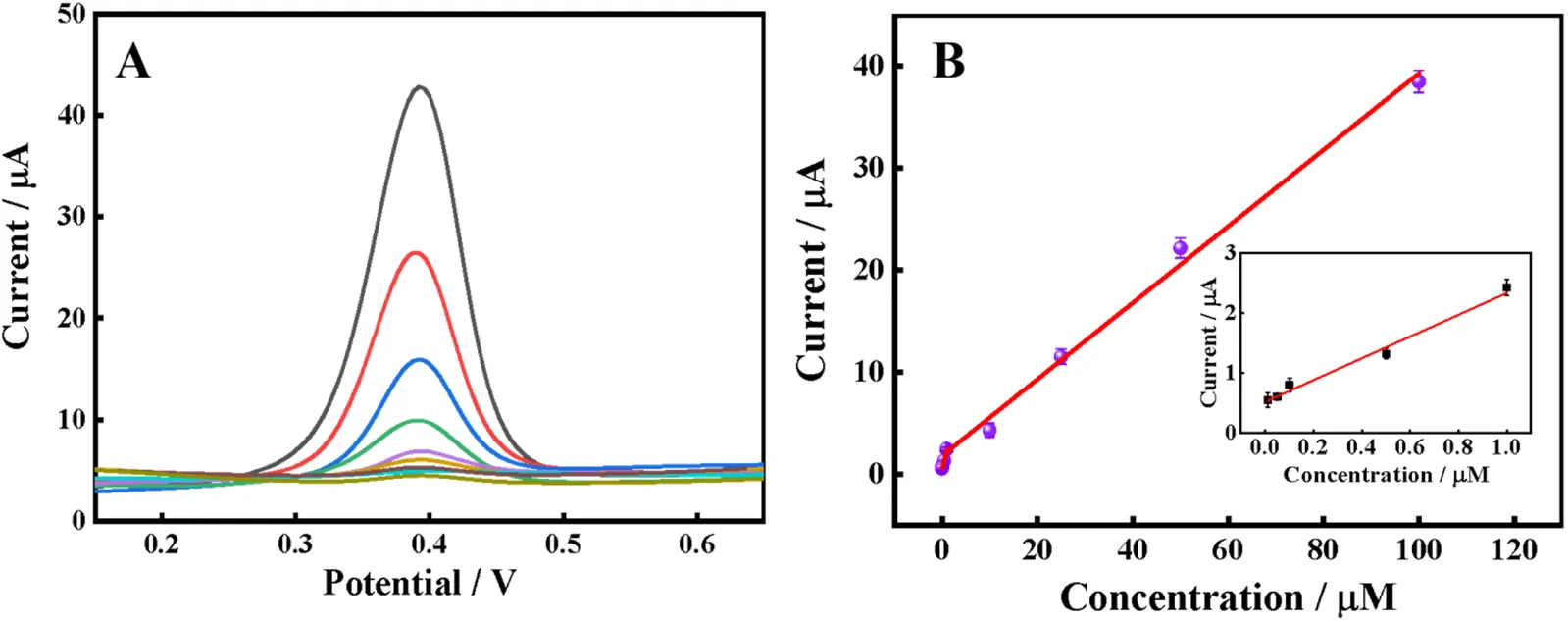
DPVs of different concentrations of rutin (0.01–100 µM) at CoNi-ZIFs/MIP/GCE sensor (A) and linear relationship between oxidation peak current and rutin concentration (B).

The calibration curve shows two linear segments with different slopes—higher at low concentrations (0.01–1 µM, 1.871 ± 0.098 µA µM^−1^) and lower at high concentrations (1–100 µM, 0.375 ± 0.017 µA µM^−1^)—and a *t*-test confirmed that the two slopes differed significantly (*P* < 0.0001). The reduced slope at higher concentrations was tentatively attributed to saturation of the specific recognition cavities: rutin first occupied the imprinted cavities (strong binding, larger current increment), and once these were saturated, additional rutin bound *via* non-specific surface adsorption on the ZIFs, whose finite number of sites gave a smaller current increment.^50,51^

In addition, the limit of detection was calculated as 2.4 nM (S/N = 3). Previously studied rutin sensors were found in Table 2 for comparison. The electrochemical sensor designed in this paper displayed wider linear range and lower detection limit.

**Table 2:** Comparison of different modified electrodes for detecting rutin

Electrode	Methods	Linear range/µM	Detection limit/nM	Ref.
CeO_2_/NiCo_2_S_4_/MWCNT/EC	DPV	0.5–65.4	43.7	10
C-GCS@ZIF-F/PL/GCE	DPV	0.1–10, 10–100	5.4	11
NMO@CNF@GCE	DPV	0.3–510	15	52
Cu_3_(HBC)_2_/SPE	DPV	0.0454–34.3088	32	53
NiO/ZnO/GCE	DPV	0.049–742.5	11	54
MIP/AuNPs-MoS_2_-GN/GCE	DPV	0.01–45	4	55
CoNi-ZIFs/MIP/GCE	DPV	0.01–1, 1 to 100	2.4	This work

### 3.7. Detection of rutin in FSI

The CoNi-ZIFs/MIP/GCE was applied to determine rutin in FSI to examine the practical application by DPV method. The standard addition method (*n* = 3) was employed for recovery evaluation. Specifically, FSI samples were spiked with rutin at three concentration levels (10, 20, and 30 µM). The calculated rutin concentrations and recovery rates obtained by the proposed DPV and HPLC methods are summarized in Table 3. To further assess the agreement between these two methods, a Bland–Altman analysis was performed based on the detection results of the four spiked levels. The analysis revealed a mean bias of 0.55 µM with 95% limits of agreement ranging from 0.11 to 0.99 µM. All data points fell well within the acceptable limits, indicating that the differences between the two methods are analytically negligible. Combined with the satisfactory recovery rates (95.7–99.3%), these results demonstrate that the CoNi-ZIFs/MIP sensor possessed high accuracy and excellent consistency with the standard HPLC method for rutin quantification. These findings demonstrated that the CoNi-ZIFs/MIP sensor can be reliably applied to the quantification of rutin in real samples.

**Table 3:** Determination of rutin in FSI samples (*n* = 3)

Spiked (µM)	DPV		HPLC	
	Founded (µM) ± SD	Recovery (%)	Founded (µM) ± SD	Recovery (%)
0	12.54 ± 0.92	—	11.83 ± 0.34	—
10	22.11 ± 1.25	95.7	21.45 ± 0.58	96.2
20	31.75 ± 1.64	96.1	31.54 ± 0.47	98.6
30	42.33 ± 1.88	99.3	41.72 ± 0.55	99.6

## 4. Conclusion

In this work, a selective electrochemical sensor for rutin detection was successfully prepared by cathodically synthesizing CoNi-ZIFs onto an MIP surface. The influence of deposition time on the electrochemical performance, morphology, and structure of CoNi-ZIFs/MIP was investigated to explore the structure–activity relationship. Compared to the previous pure MIP, which relied solely on current responses and suffered from poor potential selectivity, the *in situ* synthesis of CoNi-ZIFs on the MIP surface provided additional electrocatalytic selectivity. Consequently, both the peak potential difference (Δ*E*) and the current ratio were further enlarged, demonstrating a synergistic effect between MIP-based recognition and ZIF-based electrocatalysis to improve the selectivity toward rutin. This enhancement was likely attributable to the distinct electrocatalytic activity of CoNi-ZIFs toward different flavonoids, which might arise from the variation in the 3-position substituents on the C ring of flavonoids. The resulting sensor delivered high selectivity, sensitivity, and reliability for rutin quantification in complex samples, offering a promising strategy for flavonoid-selective electrochemical sensing.

## Author contributions

Rongqian Meng: writing – original draft, methodology, investigation, formal analysis, conceptualization, funding acquisition. Shengjuan Shao: validation, investigation, formal analysis, funding acquisition. Lijun Guo: investigation, formal analysis. Hong Yang: methodology.

## Conflicts of interest

The authors declare that they have no known competing financial interests or personal relationships that could have appeared to influence the work reported in this paper.

## Supplementary Material

RA-OLF-D6RA09585B-s001

## Data Availability

The authors confirm that the data supporting the findings of this study are available within the article and its supplementary information (SI). Supplementary information is available. See DOI: https://doi.org/10.1039/d6ra09585b.
