# Supplementary material for: Cathodic deposition of CoNi-ZIFs on molecularly imprinted polymers for synergistically enhanced electrochemical detection of rutin
Source: RSC Adv. 2026 Nov 10. Online ahead of print. doi: 10.1039/d6ra09585b (PMC13749332; doi:10.1039/d6ra09585b)
Supplement: Supplementary file 1 [file RA-OLF-D6RA09585B-s001.pdf]

Supplementary Information (SI) for RSC Advances.
This journal is © The Royal Society of Chemistry

Supporting material

Cathodic Deposition of CoNi-ZIFs on Molecularly Imprinted Polymers for Synergistically Enhanced Electrochemical Detection of Rutin

Rongqian Meng*, Shengjuan Shao, Lijun Guo, Hong Yang
Department of Chemistry and Chemical Engineering, Taiyuan Institute of Technology, Taiyuan 030008, China

2. Experimental

2.1. Materials and Instruments

2-methylimidazole (2-mIM, 99 %), Cobalt nitrate hexahydrate [$Co(NO_3)_2{\cdot}6H_2O$, 98%], nickel nitrate hexahydrate [$Ni(NO_3)_2{\cdot}6H_2O$, 98%] and Lithium perchlorate ($LiClO_4$, purity: 99%) were obtained from Rhawn Reagent Co. Ltd. (Shanghai, China), Rutin (purity: 95%), Morin (purity:>90%), Luteolin (purity: 95%), Quercetin (purity: 99%), EDOT (purity: 99%) and Pyrrole (purity: 98%) were obtained from Aladdin Biochemical Technology Co., Ltd (Shanghai,China). FSI was acquired from the local Chinese medicine market (Taiyuan, China). The stock solution of flavonoids was prepared with methanol and stored in a refrigerator at 4℃. 0.1 M Acetate Buffer Solution (ABS) prepared with acetic acid and sodium acetate was employed as flavonoids test solvent.

All the electrochemical experiments were performed on a electrochemical workstation (CHI 660E, Shanghai CH Instrument, China). A conventional three-electrode system was set up with a modified glassy carbon electrode (GCE, 3 mm diameter) as the working electrode, a Pt foil as the auxiliary electrode, and a saturated calomel electrode (SCE) as the reference electrode. The extract experiments of rutin were performed on an KQ-250DE ultrasound bath(Kunshan, China). SEM (JEOL, Japan), TEM (JEOL, Japan) and XPS (Thermo Fisher Scientific, USA) were used for morphology, crystal structure and chemical composition characterization of different materials, respectively.

2.2. Fabrication of MIP/GCE

The imprinting solution was prepared by dissolving EDOT, Py, and $LiClO_4$ in ultrapure water. Then, 1 mL of rutin stock solution (12.5 mmol/L in methanol) was pipetted into the mixture and diluted to 25 mL with ultrapure water. The final concentrations in the imprinting solution were 4.0 mM EDOT, 2.0 mM Py, 0.5 mM rutin, and 45.0 mM LiClO₄ (molar ratio of EDOT : Py : rutin = 8:4:1). The GCE was immersed and electropolymerized for 4 cycles (0～1.7 V, 0.08 V/s). After rinsing, the electrode was eluted for 30 min with NaOH/ethanol mixture (0.2 mol/L, V/V = 1:1) to remove the template, yielding MIP/GCE. The template molecules on the MIP was completely eluted if no oxidated peak of rutin appeared. For comparison, the NIPs/GCE preparation process was consistent with MIPs/GCE except for the addition of template.

2.4. Preparation of real samples

In order to evaluate the accuracy of the electrochemical method employed in the present study for the quantification of rutin in FSI, high-performance liquid chromatography (HPLC) was adopted as a reference method for validation. The chromatographic conditions for the HPLC analysis of the S. japonica bud extract were established as follows: the stationary phase comprised a SinoChrom ODS-BP column (200 mm × 4.6 mm); the detection wavelength was set at 360 nm; the column temperature was maintained at 30 ℃; and the mobile phase consisted of 0.1% phosphoric acid in water (solvent A) and acetonitrile (solvent B). Gradient elution was performed according to the following programme: 0–15 min, 15–20% B; 15–25 min, 20–35% B; 25–60 min, 35% B; and 60–70 min, 35–15% B. The flow rate was 0.6 mL/min, and the injection volume was 10 μL. Both the rutin reference standard solution and the FSI extract solution were injected separately, each in triplicate, and the rutin content in the buds was calculated using the external standard method.

3. Results and discussion

3.1. Optimization of the preparation conditions of CoNi-ZFs/MIP/GCE

As shown in Fig.S1, using the peak current of rutin as the index, the optimal preparation conditions for the CoNi-ZIFs/MIP composite were screened as follows: the Co^{2+} : Ni^{2+} molar concentration ratio of 0.1:0.1, the deposition potential of -2.0 V, and the deposition time of 250 s.

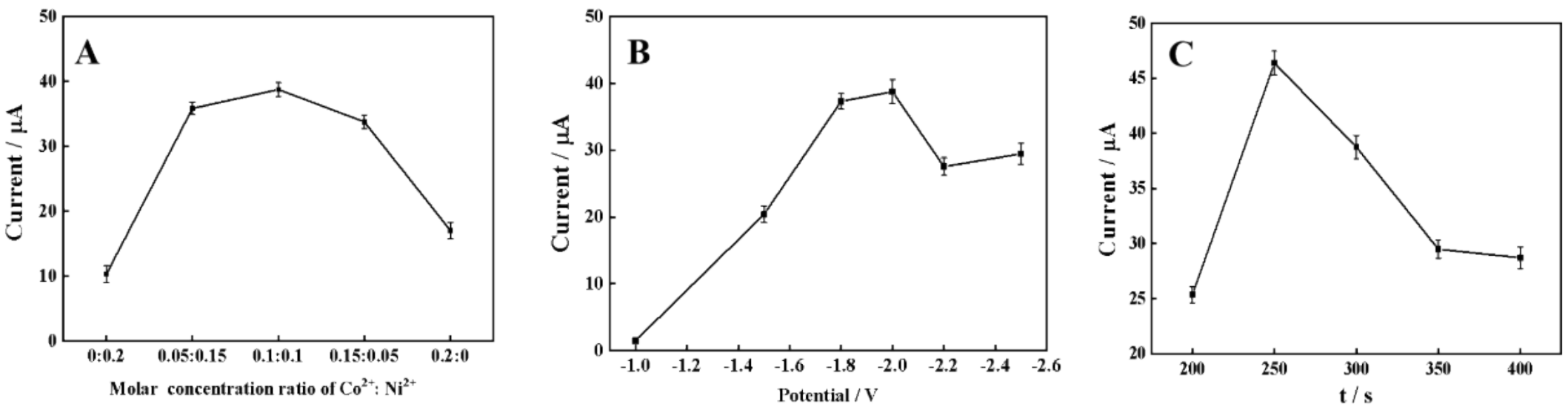

Fig.S1 The effects on rutin DPV peak current of molar concentration ratio of Co^{2+}:Ni^{2+} (A) , deposition potential (B) and deposition time(C).

3.3. Structure characterization

The XPS Binding energy and corresponding areas of Co^{3+} $2p_{1/2}$, Co^{3+} $2p_{3/2}$, Co^{2+} $2p_{1/2}$ and Co^{2+} $2p_{3/2}$ in CoNi-ZIFs-200s/MIP, CoNi-ZIFs/MIP and CoNi-ZIFs-300s/MIP were presented in the Table S1, and the area ratio of Co^{2+}/Co^{3+} were calculated[1-2]. The Binding energy and corresponding areas of Ni^{3+} $2p_{1/2}$, Ni^{3+} $2p_{3/2}$, Ni^{2+} $2p_{1/2}$ and Ni^{2+} $2p_{3/2}$ were presented in the Table S2, and the area ratio of Ni^{2+}/Ni^{3+} were calculated[1,3-4]. The N1s XPS spectrum was fitted into four peaks, corresponding to pyridinic-N (~398.5 eV), metal–N bond (~399.2 eV), pyrrolic-N (~400.4 eV), and oxidized N bonds (~406.8 eV), and the area ratios of each component were calculated(Table S3).

Table S1 Binding energy(eV) and area (CPS·eV) of Co^{3+} and Co^{2+} XPS fitting peaks in CoNi-ZIFs-200s/MIP, CoNi-ZIFs/MIP and CoNi-ZIFs-300s/MIP

	Co^{3+}				Co^{2+}				Area ratio of Co^{2+}/Co^{3+}
	$2p1/2$		$2p3/2$		$2p1/2$		$2p3/2$		
	BE	Area	BE	Area	BE	Area	BE	Area	

CoNi-ZIFs-200s/MIP	796.2	9025.43	780.7	26079.56	798.0	4381.85	782.9	9962.91	0.41
CoNi-ZIFs/MIP	796.5	10868.34	780.9	36708.77	797.8	16998.39	782.7	28598.85	0.95
CoNi-ZIFs-300s/MIP	796.3	16603.16	780.9	49893.34	797.8	17307.83	782.7	29675.69	0.71

Table S2 Binding energy(eV) and area((CPS·eV) of Ni^{3+} and Ni^{2+} XPS fitting peaks in CoNi-ZIFs-200s/MIP, CoNi-ZIFs/MIP and CoNi-ZIFs-300s/MIP

	Ni^{3+}				Ni^{2+}				Area ratio of Ni^{2+}/Ni^{3+}
	2*p*1/2		2*p*3/2		2*p*1/2		2*p*3/2		
	BE	Area	BE	Area	BE	Area	BE	Area	
CoNi-ZIFs/MIP-200s	874.5	13284.8	856.8	24892.4	872.6	26365.0	855.3	54346.94	2.11
CoNi-ZIFs/MIP	874.6	4833.3	856.9	5273.8	872.7	7951.7	855.3	16966.9	2.46
CoNi-ZIFs/MIP-300s	874.8	3219.5	857.0	4449.6	872.8	6247.79	855.4	11018.14	2.25

Table S3 Binding energy(eV) and area ratio of N 1s XPS fitting peaks in CoNi-ZIFs-200s/MIP, CoNi-ZIFs/MIP and CoNi-ZIFs-300s/MIP

	Pyridinic-N		metal-N		Pyrrolic-N		Oxidized-N/NO_3^-	
	BE	Area ratio/%	BE	Area ratio/%	BE	Area ratio/%	BE	Area ratio/%
CoNi-ZIFs-200s/MIP	398.4	32.26	399.1	26.9	400.2	35.84	406.7	5.0
CoNi-ZIFs/MIP	398.6	30.7	399.2	32.7	400.4	27.1	406.8	9.0
CoNi-ZIFs-300s/MIP	398.5	32.0	399.0	41.1	400.3	21.0	406.7	5.7

3.2. Electrochemical analysis

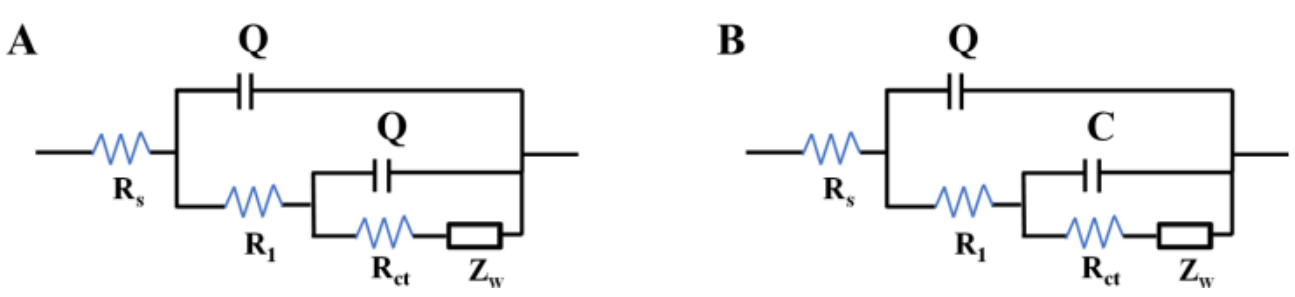

Fig.S2 (A)Fitting equivalent circuit diagrams of CoNi-ZIF-200s/MIP, (B)Fitting equivalent circuit diagrams of CoNi-ZIFs/MIP and CoNi-ZIFs-300s/MIP

Table S4 Electrochemical data for EIS curves obtained by equivalent circuit fitting

Electrode	R_s (Ω·cm²)	R_1 (Ω·cm²)	R_{ct} (Ω·cm²)	Chi-squared (▱2)
CoNi-ZIFs-200s/MIP	3.795	2.895	4113	$0.97*10^{-3}$
CoNi-ZIFs-250s/MIP	5.087	2.386	5698	$1.08*10^{-3}$
CoNi-ZIFs-300s/MIP	5.25	2.402	4760	$0.95*10^{-3}$

3.4. Selectivity analysis

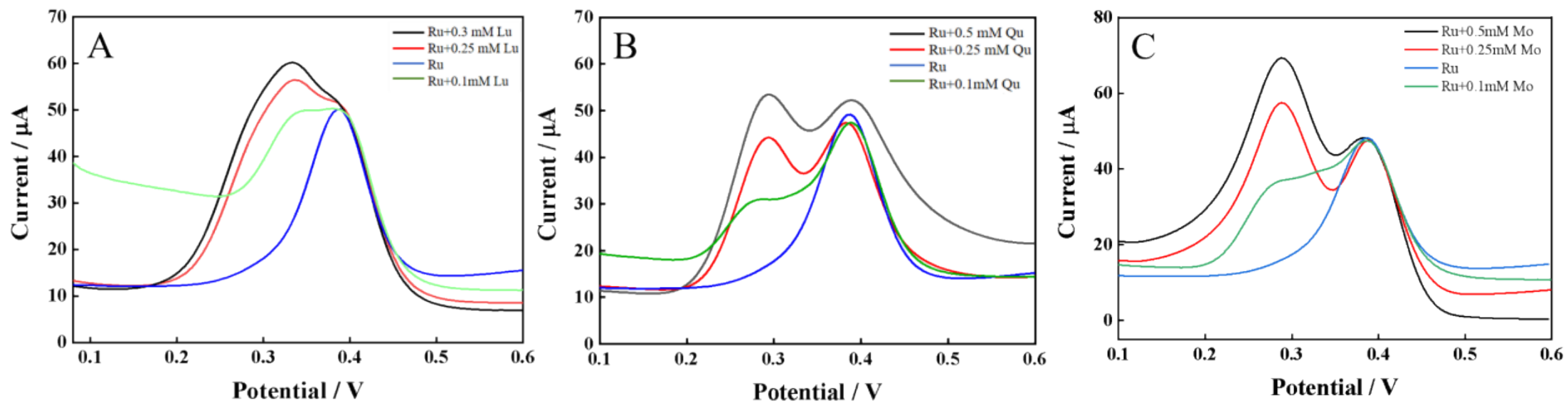

Fig. S3 DPV curves of CoNi-ZIFs/MIP/GCE in pH 4.2 ABS containing 0.1mM rutin (Ru) and varying concentrations of (A) luteolin (Lu), (B) quercetin (Qu), and (C) morin (Mo).

Table S5 The interference experiments with luteolin (Lu), quercetin(Qu), and morin (Mo) for the determination rutin.

Mixed solution	I_{Ru}/μA	RD / %	Mixed solution	I_{Ru}/μA	RD / %	Mixed solution	I_{Ru}/ μA	RD / %
Ru+0.1mM Lu	36.98	4.47	Ru+0.1mMQu	36.88	4.72	Ru+0.1mM Mo	37.21	3.87
Ru+0.25mM Lu	40.40	4.37	Ru+0.25mM Qu	37.52	3.07	Ru+0.25mM Mo	38.43	0.77
Ru+0.30Mm Lu	42.38	9.48	Ru+0.50 mMQu	35.81	7.49	Ru+0.50 mM Mo	42.23	9.09

Note: The relative deviation (RD%) was calculated as $|I_{Ru} - I^0_{Ru}| / I^0_{Ru} \times 100\%$. I_{Ru} refers to the current response of rutin in the mixed solution. (I^0_{Ru} 8.71 μA)

Reference

[1] L. Deng, X. Liu, C. Chen, L. Wang, F. Liu, J. Zhang, Ni–Co alloys via controlled pyrolysis of NiCo–MOF as heterogeneous hydrogenation catalysts, New J. Chem. 48(19) (2024) 8620-8630.

[2] H.X. Huang, S.l. Wu, C.X. Li, C.H. Wei, Study on the oxygen catalytic property of CZIF-67/NiCo2O4 composite, Ionics 30(1) (2023) 479-489.

[3] A. He, Y. Yang, S. Tao, X. Zhou, A. Wu, Q. Zhang, C. Tao, J. Du, In situ encapsulated Co3O4 nanoparticles into self-catalyzed grown CNTs for efficient CO_2 conversion, Fuel 358 (2024) 130057.

[4] X. Qian, C. Shi, M. Wan, H. Che, J. Li, Cobalt and Nickel ZIF/MXene Nanohybrids as Flame Retardants for Epoxy Polymers, ACS Appl. Nano Mater. 8(5) (2025) 2130-2140.
